# Ictal Electroencephalographic Findings in Children with Migraine Headache

**Published:** 2019

**Authors:** Hamed SHAFAGH, Mohammad Kazem BAKHSHANDEH, Esfandiar MATINI, Marjan MOHAMMADNUR, Seyedeh Mohadeseh TAHERI OTAGHSARA, Aria BAKTASH

**Affiliations:** 1 Department of Pediatrics, Faculty of Medicine, Tehran Medical sciences , Islamic Azad university ,Tehran, Iran; 2 Department of Sports Medicine, Taleghani Hospital, Shahid Beheshti University of Medical Sciences(SBMU), Tehran, Iran; 3 Department of Medicine, Tehran University of Medical Sciences(TUMS), Tehran, Iran

**Keywords:** EEG, Children, Migraine, Iran

## Abstract

**Objectives:**

In this study, the EEG findings in children with migraine headache were assessed in Bahrami Hospital, Tehran, Iran from 2014 to 2016.

**Materials & Methods:**

In this observational cross-sectional study, 71 consecutive children with migraine headache were enrolled. The EEG findings were determined and compared with other variables.

**Results:**

There were 25 cases (35.2%) with abnormal EEG and the type of EEG abnormality comprised slow waves and sharp waves in 19(68%) and 8(32%) patients, respectively

**Conclusion:**

Nearly one-third of children with migraine might have abnormal EEG.

## Introduction

Migraine headache is a common somatic pain leading to numerous problems in patients due to high frequency and recurrence (1). These are most common type of headache especially leading to seeking a physician by patients due to intermittent course (1). This would result in decreased quality of life due to mental and somatic problems (2). For this matter prompt definite diagnosis and treatment is crucial (3). More severe migraine headache would result in more decrease in quality of life (4).

Headache is also an important neurological problem in children seen in 26% of 12 to 13-yr-old and 31% of 14 to 15-yr-old subjects (5). Migraine headaches may be seen in 3 to 10 percent of children (6, 7). Diagnostic procedures are important for both diagnosis of migraine and rule out of other differential diagnoses (5-7). In migrainous patients, low excitation threshold in the region of occipital cortex may trigger epileptic seizure (8). Occipital lobe epilepsy is an important differential diagnosis of migraine headache (9). 

For this matter, electroencephalography (EEG) is an important diagnostic tool used for roll out of probable epilepsies (10). The rate of EEG abnormalities is reported from 5.4% of migraine without aura to 43.5% in children suffering from migraine with aura. 

There are little investigations on the electroencephalogram findings during the headache attacks of migraines. Hence in this study, the EEG findings in children with migraine headache were assessed.

## Materials & Methods

In this observational cross-sectional study, 71 consecutive children with migraine headache were assessed with census manner in Bahrami Children Hospital, Tehran, Iran from 2014 to 2016. 

This survey was approved by the Ethics Committee of the Tehran University of Medical Sciences. Informed consent was taken from patients and their parents. 

All cases were identified in their first visit and EEG has been performed during their headache attacks. Two pediatric neurologists evaluated the clinical data of patients and the same specialists performed electroencephalograms.

The inclusion criteria included 1) age 5-15 yr, 2) the criteria's of migraine based on the International Headache Society, 2nd Edition, First Revision; and 3) the patient at the time of headache attack. Exclusion criteria were 1) Disorders including any type of seizure, epilepsy, and encephalitis which might lead to electroencephalogram abnormalities, 2) Using any medication such as headache- prophylactic medication and antidepressant drugs; and 3) abnormal neurologic examination. 

EEG recording was in accordance with the 10-20 system. It took 20 min for every recording session including spontaneous EEG activity, intermittent photic stimulation and 3 min hyperventilation. We did not use any pre-EEG sedations. Abnormal ictal EEG defined as background suppression, focal/generalized slow EEG activity, asymmetric EEG activity, focal/generalized spike/spike- wave/polyspike/sharp waves.

The data were collected by a checklist including age, sex, family history, pain characteristics, provocation factors, relieving factors, EEG findings, and MRI results. The EEG findings in them were determined by existing medical documents and were compared according to other variables.

Data analysis was performed among 71 subjects by SPSS (version 20.0) software (Chicago, Illinois, USA). Chi-Square, Fisher, and Independent-Sample-T tests were used and were considered statistically significant at *P*-values less than 0.05.

## Results

52.1% of subjects were male. 50.7% and 49.3% were 5-9-yr-old, and 10 to 15-yr-old, respectively. The frequency of migraine headache was 5 times, 5 to 10 times, and more than 10 times in 47.9%, 43.7%, and 8.5%, respectively. The mean initiation age was 6.9 ± 1.9 year. Fifty-two patients (73.2%) had positive family history of migraine.

The pain was pulsatile, pressure, continuous, and indefinite in 4.2%, 29.6%, 7%, and 59.2%, respectively. The pain was unilateral, bilateral, occipital, orbital, and unknown in 12.7%, 39.4%, 4.2%, 35.2%, and 8.5%, respectively. The pain initiation time was at morning, whole a day, night, and at sleep in 14.1%, 43.6%, 23.9%, and 7%, respectively.

The stress, light, insomnia, and noise were the provocation factor in 80.9%, 12.6%, 40.7%, and 61.9%, respectively. Silence, sleep, analgesic, and darkroom were relieving factor in 45.5%, 39.4%, 65.2%, and 47.8%, respectively. Five patients (7%) had aura including three visual, one auditory, and one motor aura. Pain duration was less than 1 h, 1 to 2 h, and more than 2 h in 23.9%, 46.5%, and 29.6%, respectively. Nausea, phonophobia, and photophobia were seen in 65.7%, 35.2%, and 49.3%, respectively. 45.1% of children had allergy history including 11.3%, 5.6%, and 28.2% for food, drug, and respiratory allergies.

There were 25 children (35.2%) with abnormal EEG. The type of EEG abnormality comprised slow waves and sharp waves in 19 (68%) and 8 (32%) patients respectively. The region of abnormality on EEG was occipital, temporo-occipital, temporal and generalized in 12(48%), 6(24%), 2(8%) and 5(20%) cases respectively. 

Related factors for abnormal EEG were male gender (*P*=0.045) and relieving factors of headache (*P*=0.044). The MRI was done in 62% of cases.

## Discussion

In this study, the electroencephalographic findings in children with migraine headache were assessed and 35.2% had abnormal EEG as slow and sharp waves. Spike or spike waves were not seen in any of the patients. Occipital and temporo-occipital were most affected zones. Male gender and some relieving factors were contributing to EEG abnormality.

In Iran, 20% of children with migraine had abnormal EEG and the type of migraine was related to EEG abnormality (10). However, there was no association in our study. Four children with basilar migraine were assessed which all had abnormal EEG as delta and theta waves (11). However, in our study basilar migraine was not seen but the slow waves constituted the majority of abnormal EEG.

EEG should be performed in children with basilar migraine or those with prolonged aura or suspected seizure (12). However, the epilepsies and aura were not related to abnormality of EEG in our study. Twelve percent of migraine children had abnormal EEG (13). The abnormality was more common in those with shorter headache. Besides, in our study, there was no significant association. Moreover, the abnormality rate was higher.

The best time for EEG recording is at the time of a headache and during asymptomatic intervals, the diagnostic value was decreased (14). This matter explains the cause of difference in results of studies. The most diagnostic value of EEG was reported in complicated cases (15). This matter is not in congruence with our study. Hemiplegic migraine children were assessed and reported that EEG had good diagnostic value in these cases (16). Type of migraine was not related to EEG findings in our study.

**In conclusion,** nearly one-third of children with migraine might have abnormal EEG within headache attacks. However further studies with larger sample size and comparisons with other diagnostic approaches especially imaging methods are suggested.
